# Corrected goodness-of-fit index in latent variable modeling using non-parametric bootstrapping

**DOI:** 10.3389/fpsyg.2025.1562305

**Published:** 2025-03-26

**Authors:** Georgios Sideridis, Mohammed Alghamdi

**Affiliations:** ^1^ICCTR, Boston Children’s Hospital, Harvard Medical School, Boston, MA, United States; ^2^Department of Self-Development Skills, King Saud University, Riyadh, Saudi Arabia

**Keywords:** structural equation modeling, factor analysis, descriptive fit indices, model fit, small sample sizes, bootstrapping, R function

## Abstract

Latent variable modeling (LVM) is a powerful tool for validating tools and measurements in the social sciences. One of the main challenges in this method is the evaluation of model fit that is traditionally assessed using omnibus inferential statistical criteria, descriptive fit indices, and residual statistics, all of which are, to some extent, affected by sample sizes and model complexity. In the present study, an R function was created to assess fit indices after employing non-parametric bootstrapping. Furthermore, the newly proposed corrected goodness-of-fit index (CGFI) is presented as a means to overcome the abovementioned limitations. Using the data from Progress in International Student Assessment (PISA) 2022 and Progress in International Reading Literacy Study (PIRLS) 2021, the analysis of instructional leadership and the construct of bullying results revealed differential decision-making when using the present function compared to relying solely on sample estimates. It is suggested that the CGFIboot function may provide useful information toward improving our evaluative criteria in LVMs.

## 1 Introduction

Latent variable modeling (LVM) has become the primary means to test the validity of measurements comprising items to form latent constructs (Shevlin and Miles, 1998). Modeling latent variables at the measurement level involves model specification, parameter estimation, and model evaluation based on how well the constructed hypothesized model explains the variances and covariances in the observed variables. A critical discussion within the LVM literature is about identifying the appropriate evaluative criteria for judging the model fit (Pan and Liu, 2024; Sathyanarayana and Mohanasundaram, 2024; Urban and Bauer, 2021). Among the criteria are the omnibus chi-squared test and a large number of descriptive fit indices such as the root mean squared error of approximation (RMSEA; Steiger and Lind, 1980), the standardized root mean squared residual (SRMR; Jöreskog and Sörbom, 1981), the comparative fit index (CFI, Bentler, 1990), and the goodness-of-fit index (GFI; Jöreskog and Sörbom, 1982), among others. These indices are essential tools for researchers to assess how well their hypothesized models correspond to the empirical data (Thompson and Wang, 1999; Lei and Lomax, 2005); however, there is no consensus on cutoff values. Earlier recommendations suggested that fit indices should be <0.900 while more recent guidelines recommend values <0.950. The adoption of more stringent criteria followed seminal simulation studies that marked a shift from more lenient thresholds (Doğan and Özdamar, 2017; Hu and Bentler, 1999; Kline, 2015; Schermelleh-Engel et al., 2003) aimed at distinguishing “exact” and “close” fit as noted by of MacCallum et al. (1996). Other recommendations point to two-index strategies combining information from descriptive fit indices and residual statistics (e.g., Hu and Bentler, 1999). For example, one index must be the SRMR, with a value ≤8%, and the second index can be any one of the descriptive fit indices, with a value ≥0.950. Additionally, other recommendations strongly advocated in favor of using 95% confidence intervals (CIs; e.g., Browne and Cudeck, 1993). However, what confounds the picture further is not just the lack of consent but also the complexities arising from small sample sizes, actual misfit, model complexity, the presence of missing data, and so on (Marsh et al., 2004). For example, small sample sizes have been reported to be a salient problem as a majority of studies engage <200 or 100 participants (see MacCallum et al., 1999; Mundfrom et al., 2005) and consider associated problems derived from using small samples (Hogarty et al., 2005). In the present study, we aim to assist this process by providing an R function that includes a promising corrective procedure for the GFI and the inclusion of bootstrapping from the original data so that potential incidences of bias are also examined. The function is easily accessible in the link https://github.com/GS1968/CFAtools/blob/main/CGFIboot.R.

The GFI, initially introduced by Jöreskog and Sörbom (1982), has gained widespread acceptance in LVM due to its intuitive interpretation as a measure of how well the model reproduces the observed variance–covariance matrix. Its downside, however, is that, for severely misspecified models, the index tends to provide inflated estimates (Bollen and Stine, 1993; Gerbing and Anderson, 1992; Marsh et al., 1988). Some researchers consider inflation, compared to deflation, a more serious problem because they jeopardize parsimony and theoretical coherence, and tends to favor a more conservative approach (Bentler and Bonett, 1980). Concerns on distorted estimates as a function of sample size have also been reported (Gerbing and Anderson, 1992; Marsh et al., 1988). Thus, the extreme sensitivity of the GFI renders it invalid, especially in small sample studies or nested model comparisons.

Early studies to mitigate the limitations of GFI led to the development of adjusted indices, such as the adjusted goodness-of-fit index (AGFI; Jöreskog and Sörbom, 1984) and the parsimony goodness-of-fit index (PGFI; Mulaik et al., 1989), which incorporate penalties for model complexity by adjusting for the degrees of freedom and the number of estimated parameters. Despite these advancements, subsequent studies pointed out that the adjusted indices are not immune to biases, particularly when models are misspecified (Fan and Sivo, 2005; Hu and Bentler, 1998) or sample sizes are small (Shevlin and Miles, 1998). The persistent need for a more robust fit index that can effectively address the biases associated with sample size and model complexity remains a critical issue in the field. Our contribution aims to provide an easy-to-implement correction via an R function, as proposed by Wang et al. (2020), which incorporates non-parametric bootstrapping to mitigate the effects of small sample sizes.

### 1.1 A proposal for a corrective procedure

Recently, Wang et al. (2020) proposed a corrective procedure to the GFI to account for both model complexity and sample size in light of the known downward trend observed in simulation studies. Using a systematic Monte Carlo simulation by varying sample size, estimation method, model misspecification, and model complexity, by Wang et al. (2020) demonstrated that the CGFI outperformed both the GFI and its adjusted counterpart (i.e., AGFI). The CGFI was found to be more stable across varying sample sizes and more sensitive to detecting model misspecifications, thereby overcoming the shortcomings of current fit indices (Kenny and McCoach, 2003; Shevlin and Miles, 1998). This fact makes the CGFI a notable improvement over past corrections since it considers both the small-sample downward bias in all fit indices as well as the effects of model complexity in a more systematic way. While the AGFI imposes a penalty for the number of degrees of freedom used in the model and the PGFI has also been shown to have a low sensitivity to the model misspecification, it has been recommended that the CGFI was a better and more interpretable measure than these two for model fit and misspecification, particularly when used with bootstrapping techniques such as the ones employed in our analysis. Not only does this rationale further justify the need for the CGFI method, but it also clearly delineates its superiority over past GFI correction efforts, all of which support the contributions offered in this study via an easy-to-implement R function. Given that the function is based on the Lavaan package, all estimators that are available in the Lavaan package can be applied with the current CGFI function in R.

For the corrected indicator, the authors propose a cutoff estimate of 0.90 as indicative of acceptable model fit, citing the earlier cutoff criteria. However, given their reference to the CGFI to the CFI and NNFI, Non-Normative Fit Index; the revised criteria should likely be implemented with the CGFI as well (Hu and Bentler, 1999).

### 1.2 The CGFIboot function in R: description and application

To adjust for the downward bias in fit indices, Wang et al. (2020) proposed a correction factor $\frac{1}{N}$ proportional to a model’s complexity adjusting factor $\frac{k\left(k+1\right)}{p}$ to the GFI. Consequently, the corrective version of the GFI (CGFI) is defined as shown in Equation 1:


(1) $$\text{CGFI}=GFI+\frac{k\left(k+1\right)}{p}\times \frac{1}{N}$$

Where *k* is the number of observed variables, *p* is the number of free parameters, and *N* is the sample size. In LVM, the degrees of freedom and the number of free parameters (*p*) for the test model or the theoretical model $\left(df_{T}\right)$ have a verified functional relationship as follows in Equation 2:


(2) $$df_{T}=\frac{k\left(k+1\right)}{2}-p$$

Thus, an alternative form of CGFI is as shown in Equation 3:


(3) $$\text{CGFI}=GFI+\frac{2}{1-\frac{2df_{T}}{k\left(k+1\right)}}\times \frac{1}{N}$$

The corrective factor for sample size, by a factor $\frac{1}{N}$, compensates for the potentially downward bias introduced by small samples as, when N is small, $\frac{1}{N}$ is large, resulting in compensation; when *N* is large, then there is a negligible correction as large samples provide population-like estimates that are robust. Furthermore, the correction for model complexity by a factor of $\frac{k\left(k+1\right)}{p}$ accounts for the number of observed parameters relative to the number of estimated parameters. Thus, if a model is complex (having more estimated parameters than the observed variables), then the corrective procedure adjusts for model inflation; if a model is simple, the correction is small to maintain model integrity in the estimation process.

The present function encompassed the Lavaan package to estimate additional descriptive fit indices such as the CFI, TLI, Tucker-Lewis Index; GFI, AGFI, RMSEA, and SRMR. Additionally, it provides 95% confidence intervals from simulated population distributions using non-parametric bootstrapping described next. The function allows for the employment of continuous, ordered categorical and dichotomous data. Missing data are addressed via the “NA” text as the function has been designed to read comma-delimited data (.csv). The user can modify the number of replicated samples as desired. Among the estimators, the current version of Lavaan we used allowed us to apply the following estimators: ML, Maximum Likelihood; MLR, Maximum Likelihood Robust; MLM, Maximum Likelihood Mean Adjusted; WLSMV, Weighted Least Squares Mean and Variance Adjusted; DWLS, Diagonally Weighted Least Squares; GLS, Generalized Least Squares; ULS, Unweighted Least Squares; and ULSMV, Unweighted Least Squares Mean and Variance Adjusted. The availability of estimators depends on the version of Lavaan used; thus, it is not limited to the estimators mentioned here.

The non-parametric bootstrapping method involves resampling with replacement from the original sample data to form a number of pseudo-samples (bootstrap samples). These samples simulate the population distribution of a statistic, in this case, the fit indices (CGFI, CFI TLI, and RMSEA). Non-parametric bootstrapping is a particularly powerful type of resampling because it does not presume any distribution of the data, unlike parametric bootstrapping (Efron and Tibshirani, 1994), which makes this method robust to use in approximating sampling distributions when the form of an underlying population distribution does not fit a known distribution. Thus, the advantages of the procedure are its distribution-free nature, its flexibility, and its accuracy with small samples (Davison and Hinkley, 1997). The procedure’s disadvantages are computational intensity, sampling bias, where sample-based idiosyncrasies are amplified, thereby creating biased population distributions and interpretation (if distributions are severely skewed or multimodal) (Chernick, 2008). For example, with a small sample, some observations (e.g., outliers) may be replicated more frequently, reducing variability in the bootstrap samples and thus affecting the validity of the estimated parameters. Given that uncertainty is captured in the estimates of the 95% confidence intervals, non-parametric bootstrapping with small samples may result in overly optimistic (narrow) confidence intervals. Future updates of the function will include alternatives to non-parametric bootstrapping.

The output displays not only the original sample data estimates (lengthy Lavaan output) but also the averaged data from bootstrap distributions using user-defined replicated samples (1,000 in the presence instances). Discrepancies between the mean bootstrap estimates and those from the sample are likely indicative of enhanced variability, which may limit the generalization to the true population. For that purpose, standard deviations of the bootstrap distributions were added. Thus, our goal of displaying both original sample estimates and those from the bootstrap distributions is to highlight the potential concerns about findings’ generalization. As MacCallum et al. (1996) suggested, the inherent value of bootstrapping is the use of the 95% confidence intervals of the bootstrap distribution in which upper (for RMSEA) and lower bounds (for fit indices) may point to having a model with unacceptable model fit given the uncertainty introduced by the simulated population distribution.

#### 1.2.1 Example 1: evaluating the instructional leadership scale in PISA 2022

**Figure 1:**
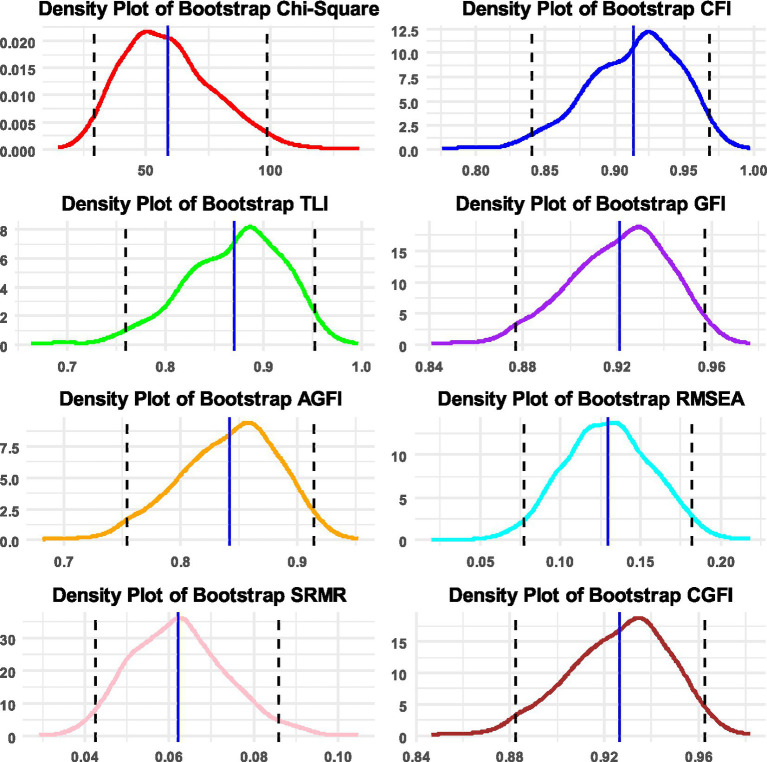
Densities of bootstrap distributions for the chi-squared statistic and the following fit indices: CFI, TLI, GFI, AGFI, RMSEA, SRMR, and CGFI, along with 95% confidence intervals. Densities are based on 1,000 replicated samples in the measurement of instructional leadership.

Using the data from the Progress in International Student Assessment (PISA 2022), the CGFIboot function was applied to the measurement of instructional leadership using a sample of 193 principals from Saudi Arabia (data are available at: https://www.oecd.org/en/about/programmes/pisa.html#data). The instrument collects data from principals who are asked how often they or another member of their school management team have participated in or conducted teaching related to instructional leadership during the past 12 months. Response scaling included five items ranging from “Never or almost never” to “Every day or almost every day.” The analysis was run with the maximum likelihood estimator with robust standard errors, which has been recommended with Likert-type data with at least five items given the adjustments on the test statistic for non-normality (Beauducel and Herzberg, 2006; Muthén and Asparouhov, 2002; Rhemtulla et al., 2012). The scale includes seven items with sample content being as follows: “How often you collaborate with teachers to solve classroom discipline problems?” or “How often you work on a professional development plan?” Internal consistency reliability was measured at 0.846 using Cronbach’s alpha and 0.850 using MacDonald’s omega. Using a principal components analysis (PCA), a single component was extracted using the eigenvalue <1 criterion, explaining 52.287% of the item’s information. The Kaiser–Meyer–Olkin (KMO) test of sampling adequacy was equal to 0.854, which is a good value for the amount of common variance making the data appropriate for use in factor analysis. Furthermore, Bartlett’s test of sphericity was also significant, suggesting that the item-factor correlation are substantial for use with the factor model [*χ*^2^(21) = 491.874, *p* < 0.001]. After applying the CGFIboot function, the results indicated model fit rejection using the omnibus chi-square test and, similarly, the descriptive fit indices assuming the more recent criterion of 0.950 (Table 1). Densities of the fit indices along with 95% confidence bands are shown in Figure 1. Interestingly, the GFI was equal to 0.945 (rejected using strict standards), but the corrective procedure resulted in a GFI estimate of 0.950. Thus, in this example, the CGFI would indicate a good fit of the data to the model, compared to the other fit indices, demonstrating a good adjustment for small sample sizes and a correction for the downward trend observed earlier (Wang et al., 2020). Additional information provided by the confidence intervals and the estimated standard deviation of the bootstrap distribution suggested that the sample may be idiosyncratic and may not generalize to a coherent population as it is highly variable. This variability is evident with the mean chi-squared estimate from the bootstrap distribution that increased from 39 to 59 units. The uncertainty of sample estimates is also evident using the 95% confidence intervals that clearly include lower estimates of fit that are not acceptable.

**Table 1:** CGFIboot function indicators for original and bootstrapped estimates in confirmatory factor analysis for the measurement of leadership in the progress in international student assessment (PISA) 2022.

Measure	Original	Boot Mean	Boot SD	CI_2.5	CI_95
Chi-Square	39.141	58.886	18.460	29.272	98.526
DF	14.000	14.000	0.000	14.000	14.000
*P*-Value	0.000	0.002	0.015	0.000	0.010
CFI	0.949	0.914	0.034	0.840	0.968
TLI	0.923	0.871	0.051	0.760	0.953
GFI	0.945	0.921	0.021	0.877	0.957
AGFI	0.889	0.842	0.043	0.754	0.914
RMSEA	0.099	0.130	0.028	0.077	0.182
SRMR	0.054	0.062	0.011	0.042	0.086
AIC	3068.588	3045.499	74.503	2896.160	3190.080
BIC	3113.444	3090.355	74.503	2941.016	3234.936
CGFI	0.950	0.926	0.021	0.882	0.963

	lhs		rhs	est	se	z	*p*-value	ci.lower	ci.upper
1	F1	=~	item1	1.000	0.000	NA	NA	1.000	1.000
2	F1	=~	item2	1.247	0.177	7.064	0.000	0.901	1.593
3	F1	=~	item3	1.402	0.223	6.276	0.000	0.964	1.840
4	F1	=~	item4	1.493	0.243	6.137	0.000	1.017	1.970
5	F1	=~	item5	1.491	0.234	6.362	0.000	1.031	1.950
6	F1	=~	item6	0.660	0.159	4.158	0.000	0.349	0.971
7	F1	=~	item7	0.990	0.212	4.664	0.000	0.574	1.407
8	item1	~~	item1	1.000	0.100	9.957	0.000	0.803	1.197
9	item2	~~	item2	0.431	0.064	6.731	0.000	0.306	0.557
10	item3	~~	item3	0.265	0.046	5.769	0.000	0.175	0.355
11	item4	~~	item4	0.269	0.051	5.278	0.000	0.169	0.369
12	item5	~~	item5	0.419	0.062	6.738	0.000	0.297	0.541
13	item6	~~	item6	0.510	0.070	7.273	0.000	0.373	0.648
14	item7	~~	item7	0.718	0.089	8.034	0.000	0.543	0.893
15	F1	~~	F1	0.288	0.093	3.102	0.002	0.106	0.470

lhs, Latent variable; rts, item name; est., estimate of factor loading; se, standard error; z, *Z*-test; ci.lower, 2.5% Confidence Interval; ci.upper, 97.5% Confidence Interval.

#### 1.2.2 Example 2: examining the psychometrics of bullying in PIRLS 2021

Using the data from the Progress in International Reading Literacy Study (PIRLS) in 2021, the bullying scale was selected for its solid psychometric properties across countries (data are available at: https://www.iea.nl/data-tools/repository/pirls). In the present study, we utilized data from 4,540 eighth graders from Saudi Arabia who were part of the 2021 cohort. The bullying scale comprised 14 items employing a frequentist-type scaling system ranging between “never” and “at least once a week.” Example items were “They said mean things about me” or “Spread lies about me.” The internal consistency of the scale was 0.895 using both Cronbach’s alpha and McDonald’s omega coefficients. Using the exploratory PCA procedure and the eigenvalue >1 criterion, a single component was extracted, explaining 43.503% of the item’s variance. The KMO test of sampling adequacy was equal to 0.946, which is a very high value for the amount of common variance making the data appropriate for use in factor analysis. Furthermore, Bartlett’s test of sphericity was also significant, suggesting that item-factor correlation is substantial for use with the factor model [*χ*^2^(91) = 23190.428, *p* < 0.001].

**Figure 2:**
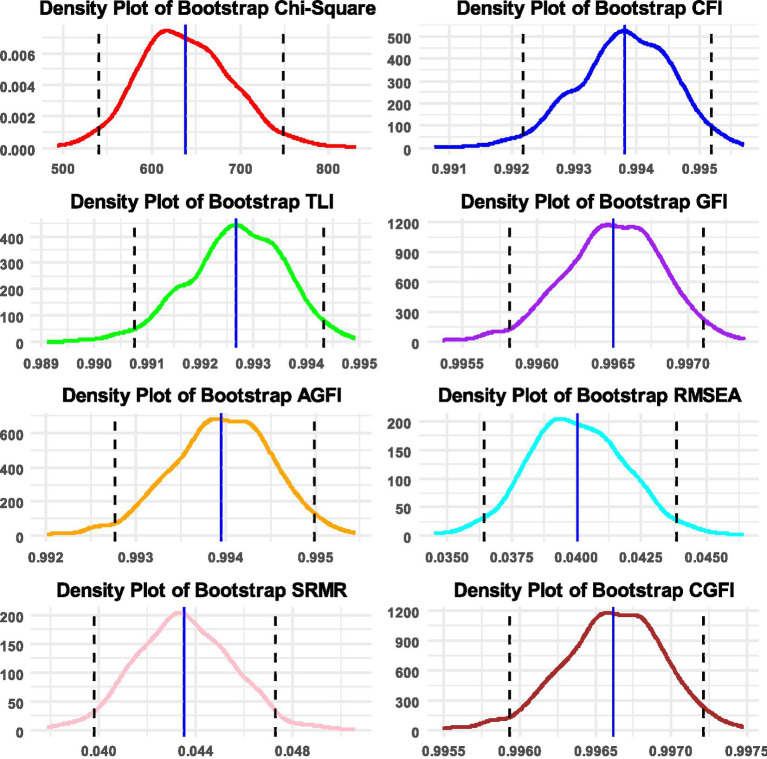
Densities of bootstrap distributions for the Chi-square statistic, and the following fit indices: CFI, TLI, GFI, AGFI, RMSEA, SRMR, and CGFI, along with 95% confidence intervals. Densities are based on using 1,000 replicated samples in the measurement of bullying.

The CGFIboot function was applied for defining ordered data and using the WLSMV estimator. Densities of the fit indices along with 95% confidence bands are shown in Figure 2 for the measurement of bullying. As shown in Table 2, the sample-based data fit the model adequately. Given the large sample size, as expected, the omnibus chi-squared test of exact fit was significant [*χ*^2^(77) = 590.326, *p* < 0.001]; therefore, preference was given to the fit indices. Among them, all were >0.950, showing an excellent fit of the data to the model. When viewing the bootstrap estimates, the results indicated that the bootstrap estimates of the descriptive fit indices were identical, with only minor deviations observed with the residual-based indices, all within the sampling error. Bootstrap confidence intervals were narrow and closely aligned with their point estimates. Therefore, for the measurement of bullying, all of the information collectively suggests good model fit and confidence that the sample-based data accurately reflect the population estimates. This finding is rather intuitive given that sample stratification and randomization procedures are closely monitored in international studies such as PIRLS.

**Table 2:** CGFIboot function indicators for original and bootstrapped estimates in confirmatory factor analysis for the measurement of bullying in PIRLS 2021.

Measure	Original	Boot Mean	Boot SD	CI_2.5	CI_95
Chi-square	590.326	638.515	52.825	540.805	749.133
DF	77.000	77.000	0.000	77.000	77.000
*p*-value	0.000	0.000	0.000	0.000	0.000
CFI	0.994	0.994	0.001	0.992	0.995
TLI	0.993	0.993	0.001	0.991	0.994
GFI	0.997	0.996	0.000	0.996	0.997
AGFI	0.994	0.994	0.001	0.993	0.995
RMSEA	0.038	0.040	0.002	0.036	0.044
SRMR	0.042	0.044	0.002	0.040	0.047
AIC	NA	NaN	NA	NA	NA
BIC	NA	NaN	NA	NA	NA
CGFI	0.997	0.997	0.000	0.996	0.997

	lhs	op	rhs	est	se	z	*p*-value	ci.lower	ci.upper
1	F1	=~	y1	0.713	0.012	61.506	0	0.691	0.736
2	F1	=~	y2	0.713	0.011	63.070	0	0.691	0.735
3	F1	=~	y3	0.698	0.012	56.288	0	0.673	0.722
4	F1	=~	y4	0.729	0.012	58.428	0	0.704	0.753
5	F1	=~	y5	0.803	0.010	76.799	0	0.782	ss0.823
6	F1	=~	y6	0.639	0.014	45.313	0	0.612	0.667
7	F1	=~	y7	0.787	0.013	62.380	0	0.762	0.812
8	F1	=~	y8	0.722	0.013	54.907	0	0.696	0.748
9	F1	=~	y9	0.804	0.012	66.970	0	0.781	0.828
10	F1	=~	y10	0.801	0.014	58.416	0	0.774	0.828
11	F1	=~	y11	0.859	0.010	86.216	0	0.840	0.879
12	F1	=~	y12	0.802	0.011	73.637	0	0.781	0.823
13	F1	=~	y13	0.771	0.012	64.751	0	0.748	0.795
14	F1	=~	y14	0.794	0.011	73.785	0	0.773	0.816
15	F1	~~	F1	1.000	0.000	NA	NA	1.000	1.000
16	y1	|	t1	−1.189	0.024	−49.000	0	−1.237	−1.142
17	y1	|	t2	−0.816	0.021	−38.784	0	−0.857	−0.775
18	y1	|	t3	−0.389	0.019	−20.336	0	−0.426	−0.351
19	y2	|	t1	−1.323	0.026	−51.048	0	−1.374	−1.272
20	y2	|	t2	−0.866	0.021	−40.525	0	−0.908	−0.824
21	y2	|	t3	−0.334	0.019	−17.597	0	−0.371	−0.297
22	y3	|	t1	−1.362	0.026	−51.485	0	−1.414	−1.310
23	y3	|	t2	−0.969	0.022	−43.739	0	−1.012	−0.925
24	y3	|	t3	−0.516	0.020	−26.427	0	−0.554	−0.478
25	y4	|	t1	−1.504	0.029	−52.434	0	−1.560	−1.448
26	y4	|	t2	−1.148	0.024	−48.185	0	−1.194	−1.101
27	y4	|	t3	−0.735	0.021	−35.754	0	−0.775	−0.694
28	y5	|	t1	−1.514	0.029	−52.466	0	−1.571	−1.458
29	y5	|	t2	−1.213	0.025	−49.429	0	−1.261	−1.165
30	y5	|	t3	−0.869	0.021	−40.633	0	−0.911	−0.828
31	y6	|	t1	−1.502	0.029	−52.428	0	−1.558	−1.446
32	y6	|	t2	−1.106	0.023	−47.284	0	−1.152	−1.060
33	y6	|	t3	−0.599	0.020	−30.167	0	−0.638	−0.560
34	y7	|	t1	−1.720	0.033	−52.084	0	−1.785	−1.655
35	y7	|	t2	−1.447	0.028	−52.166	0	−1.501	−1.392
36	y7	|	t3	−1.078	0.023	−46.631	0	−1.123	−1.033
37	y8	|	t1	−1.448	0.028	−52.176	0	−1.503	−1.394
38	y8	|	t2	−1.141	0.024	−48.053	0	−1.188	−1.095
39	y8	|	t3	−0.811	0.021	−38.619	0	−0.852	−0.770
40	y9	|	t1	−1.624	0.031	−52.497	0	−1.684	−1.563
41	y9	|	t2	−1.372	0.027	−51.582	0	−1.424	−1.320
42	y9	|	t3	−1.069	0.023	−46.417	0	−1.114	−1.024
43	y10	|	t1	−1.835	0.036	−51.083	0	−1.905	−1.765
44	y10	|	t2	−1.541	0.029	−52.524	0	−1.598	−1.483
45	y10	|	t3	−1.228	0.025	−49.687	0	−1.277	−1.180
46	y11	|	t1	−1.800	0.035	−51.439	0	−1.869	−1.732
47	y11	|	t2	−1.469	0.028	−52.289	0	−1.524	−1.414
48	y11	|	t3	−1.120	0.024	−47.604	0	−1.167	−1.074
49	y12	|	t1	−1.676	0.032	−52.324	0	−1.738	−1.613
50	y12	|	t2	−1.328	0.026	−51.111	0	−1.379	−1.277
51	y12	|	t3	−0.932	0.022	−42.625	0	−0.974	−0.889
52	y13	|	t1	−1.685	0.032	−52.282	0	−1.748	−1.621
53	y13	|	t2	−1.362	0.026	−51.485	0	−1.414	−1.310
54	y13	|	t3	−0.908	0.022	−41.888	0	−0.950	−0.866
55	y14	|	t1	−1.671	0.032	−52.344	0	−1.734	−1.608
56	y14	|	t2	−1.316	0.026	−50.967	0	−1.367	−1.266
57	y14	|	t3	−0.828	0.021	−39.195	0	−0.869	−0.786
58	y1	~~	y1	0.491	0.000	NA	NA	0.491	0.491
59	y2	~~	y2	0.492	0.000	NA	NA	0.492	0.492
60	y3	~~	y3	0.513	0.000	NA	NA	0.513	0.513
61	y4	~~	y4	0.469	0.000	NA	NA	0.469	0.469
62	y5	~~	y5	0.355	0.000	NA	NA	0.355	0.355
63	y6	~~	y6	0.591	0.000	NA	NA	0.591	0.591
64	y7	~~	y7	0.380	0.000	NA	NA	0.380	0.380
65	y8	~~	y8	0.479	0.000	NA	NA	0.479	0.479
66	y9	~~	y9	0.353	0.000	NA	NA	0.353	0.353
67	y10	~~	y10	0.358	0.000	NA	NA	0.358	0.358
68	y11	~~	y11	0.262	0.000	NA	NA	0.262	0.262
69	y12	~~	y12	0.357	0.000	NA	NA	0.357	0.357
70	y13	~~	y13	0.405	0.000	NA	NA	0.405	0.405
71	y14	~~	y14	0.369	0.000	NA	NA	0.369	0.369
72	y1	~*~	y1	1.000	0.000	NA	NA	1.000	1.000
73	y2	~*~	y2	1.000	0.000	NA	NA	1.000	1.000
74	y3	~*~	y3	1.000	0.000	NA	NA	1.000	1.000
75	y4	~*~	y4	1.000	0.000	NA	NA	1.000	1.000
76	y5	~*~	y5	1.000	0.000	NA	NA	1.000	1.000
77	y6	~*~	y6	1.000	0.000	NA	NA	1.000	1.000
78	y7	~*~	y7	1.000	0.000	NA	NA	1.000	1.000
79	y8	~*~	y8	1.000	0.000	NA	NA	1.000	1.000
80	y9	~*~	y9	1.000	0.000	NA	NA	1.000	1.000
81	y10	~*~	y10	1.000	0.000	NA	NA	1.000	1.000
82	y11	~*~	y11	1.000	0.000	NA	NA	1.000	1.000
83	y12	~*~	y12	1.000	0.000	NA	NA	1.000	1.000
84	y13	~*~	y13	1.000	0.000	NA	NA	1.000	1.000
85	y14	~*~	y14	1.000	0.000	NA	NA	1.000	1.000
86	y1	~1	0.000	0.000	NA	NA	0.000	0.000	
87	y2	~1	0.000	0.000	NA	NA	0.000	0.000	
88	y3	~1	0.000	0.000	NA	NA	0.000	0.000	
89	y4	~1	0.000	0.000	NA	NA	0.000	0.000	
90	y5	~1	0.000	0.000	NA	NA	0.000	0.000	
91	y6	~1	0.000	0.000	NA	NA	0.000	0.000	
92	y7	~1	0.000	0.000	NA	NA	0.000	0.000	
93	y8	~1	0.000	0.000	NA	NA	0.000	0.000	
94	y9	~1	0.000	0.000	NA	NA	0.000	0.000	
95	y10	~1	0.000	0.000	NA	NA	0.000	0.000	
96	y11	~1	0.000	0.000	NA	NA	0.000	0.000	
97	y12	~1	0.000	0.000	NA	NA	0.000	0.000	
98	y13	~1	0.000	0.000	NA	NA	0.000	0.000	
99	y14	~1	0.000	0.000	NA	NA	0.000	0.000	
100	F1	~1	0.000	0.000	NA	NA	0.000	0.000	

lhs, Latent variable; rts, item name; est., estimate of factor loading; se, standard error; z, *Z*-test; ci.lower, 2.5% Confidence Interval; ci.upper, 97.5% Confidence Interval.

## 2 Conclusion and future directions

Overall, the CGFIboot function represents a valuable solution to some of the limitations inherent to the use of classic fit indices applied within the LVM framework, especially with small samples and complex models. With the use of a correction factor and non-parametric bootstrapping to calculate bias corrected confidence intervals (CIs) for CGFI, the results from its application to both small and large sample datasets showed the robustness of the CGFI.

While the CGFI represents a significant improvement over traditional goodness-of-fit measures, particularly in addressing sample size sensitivity and model complexity, it is not without its limitations. Although the CGFI introduces a correction factor for model complexity, its performance under very large models with high-dimensional structures needs further empirical validation. In high-dimensional SEM models (e.g., involving multiple latent constructs, hierarchical structures, or many free parameters), the correction factor applied in CGFI may not fully compensate for the distortions caused by model complexity. In simulation studies, Wang et al. (2020) demonstrated that CGFI performed well across a range of model complexities. However, for models with a very high number of parameters relative to the degrees of freedom, the index might not penalize complexity as strongly as expected, leading to inflated fit estimates. Furthermore, the model has not been tested with longitudinal designs and nested data as additional complexity is introduced with variability in intraclass correlation coefficients and the presence of model constraints. Therefore, we recommend, as is customary, that the CGFI is not used in isolation but can be combined with other descriptive fit indices, such as the CFI, RMSEA, and SRMR, which may better capture the aspects of model fit that CGFI alone cannot detect. For example, in models where high correlations among latent variables exist, CGFI might overestimate model fit, while RMSEA and SRMR might indicate significant model misfit. While the results highlight the function’s potential to enhance our “conclusion validity,” it is important to conduct further testing with diverse datasets and across a wider range of contexts, including different types of data, model complexities, and sample sizes, to assess its robustness and generalizability. Comparative studies in which the CGFI is evaluated with respect to other emerging fit indices under a variety of conditions could offer important information about its strengths and weaknesses. In addition, extending the scope of this function to longitudinal and multi-group LVM models might provide new avenues for fit evaluation in more complex research designs. Finally, to improve method transparency and replicability in LVM, we may need to develop more user-friendly interfaces/packages that would enable researchers to easily implement the present R function, which is a step in the right direction.

## Data Availability

Publicly available datasets were analyzed in this study. This data can be found at: https://www.iea.nl/data-tools/repository/pirls and https://www.oecd.org/en/about/programmes/pisa.html#data.

## Appendix A

### Examples using the CGFI function in R

Example 1: Bullying

# Load the data.

data <−read.csv(“data.csv”).

# Define your LVM model in Lavaan.

model <− “F1 = ~ NA*y1 + y2 + y3 + y4 + y5 + y6 + y7 + y8 + y9 + y10 + y11 + y12 + y13 + y14.

F1 ~ ~ 1*F1.”

# Call the function.

perform_cfa_with_bootstrap(data, model, estimator = “WLSMV,” data_type = “ordered,” n_bootstrap = 1,000).

Example 2: Instructional leadership

# Load the data.

data <− read.csv(“data.csv,” header = TRUE).

# Define your LVM model in Lavaan.

model <− “F1 = ~ item1 + item2 + item3 + item4 + item5 + item6 + item7.”

# Call the function.

perform_cfa_with_bootstrap(data, model, estimator = “MLR,” data_type = “continuous,” n_bootstrap = 1,000).

## References

[ref1] Beauducel A. Herzberg P. Y. (2006). On the performance of maximum likelihood versus means and variance adjusted weighted least squares estimation in CFA. Struct. Equ. Model. Multidiscip. J. 13, 186–203. doi: 10.1207/s15328007sem1302_2

[ref2] Bentler P. M. Bonett D. G. (1980). Significance tests and goodness of fit in the analysis of covariance structures. Psychol. Bull. 88, 588–606. doi: 10.1037/0033-2909.88.3.588

[ref001] Bentler P. M. (1990). Comparative Fit Indexes in Structural Models. Psychological Bulletin, 107, 238–246. doi: 10.1037/0033-2909.107.2.238 2320703

[ref3] Bollen K. A. Stine R. A. (1993). Bootstrapping goodness-of-fit measures in structural equation models. Sociol. Methods Res. 21, 205–229. doi: 10.1177/0049124192021002004, PMID: 40066071

[ref4] Browne M. W. Cudeck R. (1993). “Alternative ways of assessing model fit” in Testing structural equation models. eds. Bollen K. A. Long J. S. (London: SAGE Publications), 136–162.

[ref5] Chernick M. R. (2008). Bootstrap methods: A guide for practitioners and researchers. Hoboken, NJ: John Wiley & Sons.

[ref6] Davison A. C. Hinkley D. V. (1997). Bootstrap methods and their application. Cambridge: Cambridge University Press.

[ref7] Doğan I. Özdamar K. (2017). The effect of different data structures, sample sizes on model fit measures. Commun. Stat. Simulation Comput. 46, 7525–7533. doi: 10.1080/03610918.2016.1241409

[ref8] Efron B. Tibshirani R. J. (1994). An introduction to the bootstrap. London: CRC Press.

[ref9] Fan X. Sivo S. A. (2005). Sensitivity of fit indices to misspecified structural or measurement model components: rationale of two-index strategy revisited. Struct. Equ. Model. 12, 343–367. doi: 10.1207/s15328007sem1203_1

[ref10] Gerbing D. W. Anderson J. C. (1992). Monte Carlo evaluations of goodness of fit indices for structural equation models. Sociol. Methods Res. 21, 132–160. doi: 10.1177/0049124192021002002

[ref11] Hogarty K. Y. Hines C. V. Kromrey J. D. Ferron J. M. Mumford K. R. (2005). The quality of factor solutions in exploratory factor analysis: the influence of sample size, communality, and over determination. Educ. Psychol. Meas. 65, 202–226. doi: 10.1177/0013164404267287

[ref12] Hu L. T. Bentler P. M. (1998). Fit indices in covariance structure modeling: sensitivity to under parameterized model misspecification. Psychol. Methods 3, 424–453. doi: 10.1037/1082-989X.3.4.424

[ref13] Hu L. T. Bentler P. M. (1999). Cutoff criteria for fit indexes in covariance structure analysis: conventional criteria versus new alternatives. Struct. Equ. Model. Multidiscip. J. 6, 1–55. doi: 10.1080/10705519909540118

[ref14] Jöreskog K. G. Sörbom D. (1981). LISREL V: analysis of linear structural relationships by the method of maximum likelihood. Chicago, IL: National Educational Resources.

[ref15] Jöreskog K. G. Sörbom D. (1982). Recent developments in structural equation modeling. J. Mark. Res. 19, 404–416. doi: 10.1177/002224378201900402

[ref16] Jöreskog K. G. Sörbom D. (1984). LISREL VI: analysis of linear structural relationships by the method of maximum likelihood. Chicago, IL: National Educational Resources.

[ref17] Kenny D. A. McCoach D. B. (2003). Effect of the number of variables on measures of fit in structural equation modeling. Struct. Equ. Model. 10, 333–351. doi: 10.1207/S15328007SEM1003_1

[ref18] Kline R. B. (2015). Principles and practice of structural equation modeling. 4th Edn. London: Guilford Press.

[ref19] Lei M. Lomax R. G. (2005). The effect of varying degrees of non-normality in structural equation modeling. Struct. Equ. Model. 12, 1–27. doi: 10.1207/s15328007sem1201_1

[ref20] MacCallum R. C. Browne M. W. Sugawara H. M. (1996). Power analysis and determination of sample size for covariance structure modeling. Psychol. Methods 1, 130–149. doi: 10.1037/1082-989X.1.2.130

[ref21] MacCallum R. C. Widaman K. F. Zhang S. Hong S. (1999). Sample size in factor analysis. Psychol. Methods 4, 84–99. doi: 10.1037/1082-989X.4.1.84 26822184

[ref22] Marsh H. W. Balla J. R. McDonald R. P. (1988). Goodness-of-fit indexes in confirmatory factor analysis: the effect of sample size. Psychol. Bull. 103, 391–410. doi: 10.1037/0033-2909.103.3.391

[ref23] Marsh H. W. Hau K. T. Wen Z. (2004). In search of golden rules: comment on hypothesis-testing approaches to setting cutoff values for fit indexes and dangers in overgeneralizing Hu and Bentler’s (1999) findings. Struct. Equ. Model. Multidiscip. J. 11, 320–341. doi: 10.1207/s15328007sem1103_2

[ref24] Mulaik S. A. James L. R. Van Alstine J. Bennett N. Lind S. Stilwell C. D. (1989). Evaluation of goodness-of-fit indices for structural equation models. Psychol. Bull. 105, 430–445. doi: 10.1037/0033-2909.105.3.430

[ref25] Mundfrom D. J. Shaw D. G. Ke T. L. (2005). Minimum sample size recommendations for conducting factor analyses. Int. J. Test. 5, 159–168. doi: 10.1207/s15327574ijt0502_4

[ref26] Muthén B. Asparouhov T. (2002). Latent variable analysis with categorical outcomes: multiple-group and growth modeling in Mplus. Mplus Web Notes 4, 1–22.

[ref27] Pan F. Liu Q. (2024). Evaluating fit indices in multilevel latent growth models with unbalanced designs. Front. Psychol. 15:1366850. doi: 10.3389/fpsyg.2024.1366850/full, PMID: 38765833 PMC11100987

[ref28] Rhemtulla M. Brosseau-Liard P. É. Savalei V. (2012). When can ordinal variables be treated as continuous? A comparison of robust continuous and categorical SEM estimation methods. Psychol. Methods 17, 354–373. doi: 10.1037/a0029315, PMID: 22799625

[ref29] Sathyanarayana S. Mohanasundaram T. (2024). Fit indices in structural equation modeling and confirmatory factor analysis: reporting guidelines. Asian J. Econ. Bus. Account. 24, 561–577. doi: 10.9734/ajeba/2024/v24i71430, PMID: 40066690

[ref30] Schermelleh-Engel K. Moosbrugger H. Müller H. (2003). Evaluating the fit of structural equation models: tests of significance and descriptive goodness-of-fit measures. Methods Psychol. Res. Online 8, 23–74.

[ref31] Shevlin M. Miles J. N. V. (1998). Effects of sample size, model specification, and factor loadings on the GFI in confirmatory factor analysis. Personal. Individ. Differ. 25, 85–90. doi: 10.1016/S0191-8869(98)00055-5

[ref32] Steiger J. H. Lind J. C. (1980). “Statistically based tests for the number of common factors,” in *Paper Presented at the Annual Meeting of the Psychometric Society*, Iowa City, IA.

[ref33] Thompson B. Wang L. (1999). Effects of sample size, estimation methods, and model specification on structural equation modeling fit indexes. Struct. Equ. Model. 6, 56–83. doi: 10.1080/10705519909540119, PMID: 40051498

[ref34] Urban C. J. Bauer D. J. (2021). Deep learning-based estimation and goodness-of-fit for large-scale confirmatory item factor analysis. Psychometrika 86, 873–900. doi: 10.1007/s11336-021-09800-0 33528784

[ref35] Wang K. Xu Y. Wang C. Tan M. Chen P. (2020). A corrected goodness-of-fit index (CGFI) for model evaluation in structural equation modeling. Struct. Equ. Model. 27, 735–749. doi: 10.1080/10705511.2019.1695213

